# Dental emergency care in Spain during the state of alarm due to COVID-19 pandemic

**DOI:** 10.4317/jced.58064

**Published:** 2021-09-01

**Authors:** Nuria Escribano, Isabel Giráldez, Laura Ceballos, Fátima Cerdán, Raquel Infante, María-Victoria Fuentes

**Affiliations:** 1 PhD, DDS, Assistant Professor, IDIBO Research Group, Health Sciences Faculty, University Rey Juan Carlos, Alcorcón, Madrid, Spain; 2 PhD, DDS, Professor, IDIBO Research Group, Health Sciences Faculty, University Rey Juan Carlos, Alcorcón, Madrid, Spain; 3 DDS. PhD Degree Student, Health Sciences Faculty, University Rey Juan Carlos, Alcorcón, Madrid, Spain; 4 PhD Candidate, Private Dental Practice, Madrid, Spain; 5 PhD, DDS, Associate Professor, IDIBO Research Group, Health Sciences Faculty, University Rey Juan Carlos, Alcorcón, Madrid, Spain

## Abstract

**Background:**

The first state of alarm due to COVID-19 in Spain led to limit dental treatment exclusively to emergencies. The objective of the survey was to evaluate the amount and type of emergencies attended during this period, as well as to know how they were solved, and what measures were adopted to carry out dental care in these exceptional circumstances.

**Material and Methods:**

This cross-sectional study included 312 Spanish dentists, who fulfilled an online questionnaire with 22 closed questions, divided into five sections regarding to various aspects of professional dental profile and emergency care. Descriptive statistic and Chi-square tests were performed (*p*<0.05).

**Results:**

75.64% of respondents attended emergencies in person only when required, being dental pain the main emergency (90.38%). Dental emergency care in person involved a prior telephone triage of the patient to identify possible COVID-19 symptoms, as well as protective measures implementation for both, the patient and the dentist, at dental office.

**Conclusions:**

The number of dental emergencies decreased during the state of alarm, being dental pain the main cause of dental assistance via telephone or in person. Triage of patients before scheduling an in-person appointment and protective measures implementation were common features in dental emergency care during the first state of alarm period.

** Key words:**Dental emergencies, COVID-19, state of alarm, survey.

## Introduction

Severe acute respiratory syndrome coronavirus 2 (SARS-CoV-2) was officially announced as the causative pathogen of COVID-19 by the Chinese Center for Disease Control and Prevention on January 2020 (1). First cases were reported in Wuhan, China, and were identified as “Pneumonia of unknown etiology” in December 2019. On January 2020, the World Health Organization (WHO) declared the rampant spread of SARS-CoV-2 and its associated disease (COVID-19) a public health emergency with a currently known overall mortality rate to be as high as 3.4% (2). The first two cases in Spain were diagnosed on 11th February 2020 (3) and only one month later, 2128 COVID-19 cases and 47 deaths had been reported (4). This dramatic increase in cases and deaths forced the Government of Spain to declare a state of alarm on 13th March, due to begin on 14th March (5), which implied the lockdown of the entire population, the closure of educational, sports, commercial and leisure centers, as well as the suspension of most professional activities.

The closure of dental offices was not mandatory by the Government of Spain during the first state of alarm (6). On their own accord, many dental professionals postponed elective dental treatments, providing only emergency dental services, abiding by the General Council of Dentists of Spain recommendations (7). Others decided to simply close their dental offices and not carry out any kind of face-to-face treatment. Dental professionals are at high risk for nosocomial infection and can become potential carriers of the disease. These risks can be attributed to the aerosol generation, handling of sharps, and proximity to the patient’s oropharyngeal region (8). In addition, if adequate precautions are not taken, the dental office can potentially expose patients to cross contamination (8).

On 15th April, only one month later, the General Council of Dentists of Spain published a “Safety Guidance for Dental Emergency Care” (9), like other countries national dental associations (10). This document firstly defined what was considered a dental emergency, following American Dental Association guidelines (11). Secondly, telephone triage was suggested, as recommended by Ather *et al*. (8). This initial screening via telephone was intended to identify patients with suspected or possible COVID-19 infection at the time of scheduling appointments and also to assess whether the patient had a true dental emergency. The triage should include questions about any exposure to COVID-19 or presence of any symptoms of febrile respiratory illness such as fever or cough. In third place, the General Council of Dentists of Spain’s Statement proposed several measures against cross-infection and specific dental treatment recommendations based on previously published guidelines (8,12).

Therefore, during these days several circumstances concurred that made this situation being a great challenge for both, dental professionals and patients. The uncertainty generated due to the epidemiological situation, with days counting more than 800 deaths, was intensified by the absence of a precise guide to attend dental patients from the beginning of the first state of alarm. The lack of personal protective equipment (PPE) was another difficulty, because in the first days of the pandemic they were massively donated by dentists to hospitals and later there were shortage. And, of course, the fear of dentists and patients to become infected with collapsed hospitals were some of them. This people’s fear of COVID-19, because of its novel and rapid transmission, made them reluctant to go to public places including dental offices, except in an emergency (12).

As far as we know, there is no available data about the features of the dental care provision in Spain during the most restrictive period of the state of alarm, from 14th March to 4th May. Thereupon, the aim of this survey was to report the amount and characteristics of dental treatments performed during this period in Spain, as well as the patient management and protection measures adopted by dental professionals against COVID-19 at dental office.

## Material and Methods

The present cross-sectional study used an online survey questionnaire available from 27th April to 4th May 2020, designed by expert study participants at Google survey forms. Previously, a pilot test among trusted partners was carried out before sending the survey.

The online survey link circulated through social media and addressed to dentists actively working in Spain, maintaining the anonymity of the participants and thanking them for their responses. The survey could only be completed by those participants who consented to be part of the study and who had attended dental emergencies since the declaration of the state of alarm (14th March), in person or by phone.

The questionnaire, which can be accessed using the following DOI https://doi.org/10.21950/3STT2Q, was comprised of a total of 22 closed-ended questions, some of which allowed multiple responses, divided into five sections. The first section was focused on descriptive questions about sociodemographic and professional data of the participant, such as gender, group of age, dental expertise, type of dental office, number of inhabitants of the town where the office was located, number of surgeries in the dental office and dental expertise. The second section asked if the participant had attended emergencies by telephone or in person during the state of alarm by COVID-19, and generic information about emergencies. The questionnaire’s third section inquired about the telephone management of dental emergencies while the fourth section was focused on management of dental emergencies in person, and patient management and protection measures adopted for dental emergency care. In both sections, questions focused on whether the number of emergencies had increased during this period and what type of emergencies occurred. The last part of the survey gathered information about the implemented measures in their professional activity to face dental emergencies during COVID-19 outbreak.

Statistical analyses were performed using Stata 16 Software (StataCorp, College Station, TX, USA). A descriptive statistic of the responses obtained was performed, expressing the results as percentages. Some of the multi-answer questions were coded as binary variables (yes / no). Chi-square tests were applied to determine the association between sociodemographic and professional profile questions about the participant and other variables. A *p* value of <0.05 was accepted as statistically significant.

## Results

This survey was completed by 312 dentists, those who attended dental emergencies during the state of alarm out of a total of 346, from all regions across the country. Within them, 4.49% did not answer one of the questions and 0.64% did not answer two. These questions without answers were not taken into account in the descriptive statistics. Moreover, statistically significant results will be preferably highlighted.

-*Sociodemographic and dental expertise data*

The information regarding these questions is detailed in Table 1. The respondents were mainly women (66.35%) with an age between 30-49 years (70.51%), general dental practitioners (49.36%), working in their own private dental office (67.52%), with one or two dental surgeries (50.32%) in urban areas (62.82%).

**Table 1:**
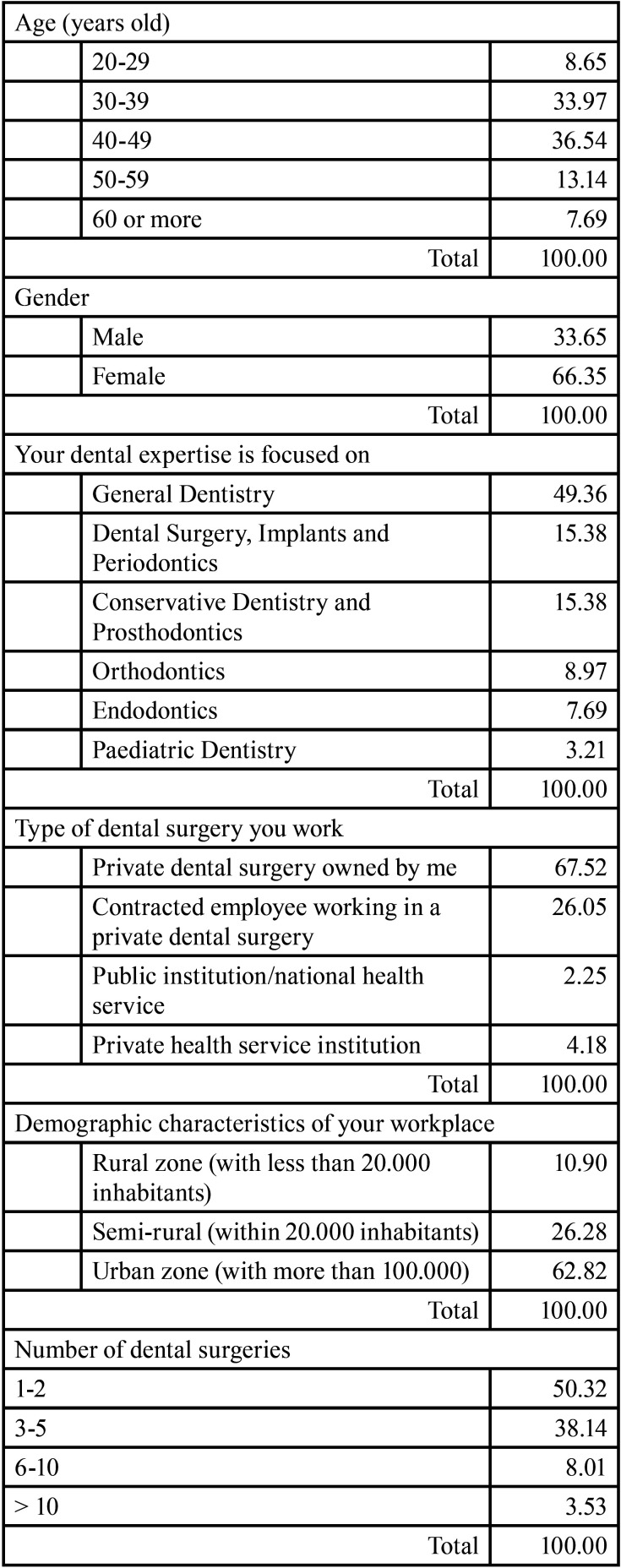
Demographic data (in percentage).

-*Generic information regarding dental emergencies care*

Most of the dentist (75.64%) informed that were attending emergencies in person only when it was required, 19.23% exclusively by phone, and 5.13% were attending their dental offices in their usual schedule. Age was significantly related (*p*=0.037) to the emergency dental care provided, as dentists over 60 years of age opted for telephone assistance, meanwhile the rest of the age groups opted for face-to-face attention. There was a significant relationship between the type of dental office and how the emergency dental care was provided (*p*<0.001), as dentists working in public healthcare institutions had attended emergencies in person in most cases within their usual schedule (57.14%). Meanwhile in private dental offices of its own ownership (81.43%), owned by others (69.14%), or in private healthcare institutions (61.54%) most of dentists attended only in person only when it was essential.

The majority of the respondents (65.06%) considered that the number of emergencies had diminished during the state of alarm, while 23.72% perceived that was as usual, and 11.22% noted an increase.

They were also asked about the reasons why patients contacted their dental offices and the main emergency was related to pain (90.38%), followed by restoration fracture or loss (39.74%) and orthodontic problems (28.21%). Detailed information regarding the answers is shown in Table 2. These three main emergencies were related to dental expertise (*p*<0.001, *p*=0.003, *p*=0.01, respectively). Pain was the main emergency answered by 100% of the endodontists and 93% of the general practitioners.

**Table 2:**
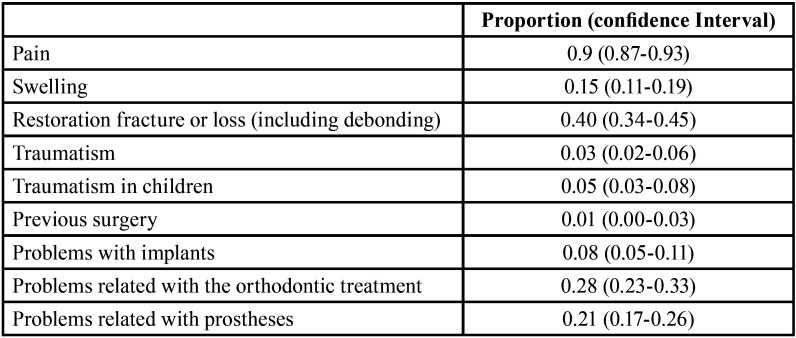
Reasons to contact the dental office (proportion).

Regarding how dentists had mainly solved the emergencies, most were able to resolve them by telephone prescribing antibiotics, analgesics and anti-inflammatories (59.94%), followed by those who had to attend the emergency in person (26.60%) and thirdly, by those that could solve it exclusively by phone (13.46%). There was a significant relationship with gender (*p*=0.026), being women capable of managing emergencies not in person in a higher percentage.

Concerning the relationship between the location of the dental office and the way in which emergencies were resolved, there was a statistically significant relationship (*p*=0.041). Most of the emergencies were solved by phone with prescription of medications in rural areas (75.53%), semi-urban (67.07%) and rural areas (54.59%). However, the resolution only via telephone was higher in urban areas (16.84%), in comparison with semi-urban (9.76%) and rural areas (2.94%). And, also face-to-face care, with a percentage of 28.57% in urban areas and 23.71% for semi-urban areas and 23.53% for rural areas.

Finally, regarding dental expertise, emergencies were mostly resolved by telephone with the prescription of antibiotics or anti-inflammatories, except in Orthodontics, where problems were resolved by telephone (46.43%), followed by prescription (22.14%) and in person (21.43%).

-*Telephone management of dental emergencies*

All participants were specifically asked about the number of emergencies that had been solved by phone weekly, and 45.51% answered less than 5, 37.82% between 5 and 10, and 16.67% more than 10.

-*Management of dental emergencies in person*

Regarding emergencies that had to be attended in person, 21.15% of the dentists declared that no emergency required to be treated personally, 46.15% attended less than 5 a week, 24.68% between 5 and 10 and 8.01% more than 10.

Dentists were also asked about the most frequent emergency that needed to be treated in person according to those described by the ADA (11). The main reason was “severe dental pain” (69.07%), followed by any other emergency that after phone triage was considered to require attention in person (36.86%), intra-oral or extra-oral swelling that potentially compromise patient’s airways (19.07%), other emergencies (5.51%), trauma (4.24%) and bleeding (1.27%).

-*Patient management and protection measures adopted for dental emergency care in person*

Dentists were asked in a multiple-choice question about the initial screening made via telephone. The most frequent choice (35.95%) combined questions regarding fever and/or breathing problems, other signs and symptoms related with COVID-19, being in contact with a known or suspected COVID-19 patient, and being a high risk professional, followed by the combination of the first three (35.54%).

They were also asked regarding the instructions provided to patients before attending the face-to-face appointment, in a multiple-choice question. The most frequent combination of instructions (48.57%) included to arrive at the agreed time, attend alone, with mask and keeping safe distance.

Regarding the preventive measures adopted when the patient arrives to the dental office and before being attended, all participants indicated that they asked patients to wash their hands with an alcohol-based hand rub.

Dentists were also asked if they were attending emergencies on their own, and most dentist answered yes (44.93%), while 30.40% worked four-handed, although not all the time and 24.67% worked four-handed always.

Regarding questions about preventive measures implemented in the dental surgery to avoid cross-infection during the first state of alarm (Table 3), the most outstanding answers were the avoidance of use of high-speed dental handpiece (55.45%), the protection of inanimate surfaces with disposable plastic covers (51.28%), and room air renewal after each patient (41.35%). A remarkable percentage of dentists (41.99 %) continued to use their usual disinfection protocols.

**Table 3:**
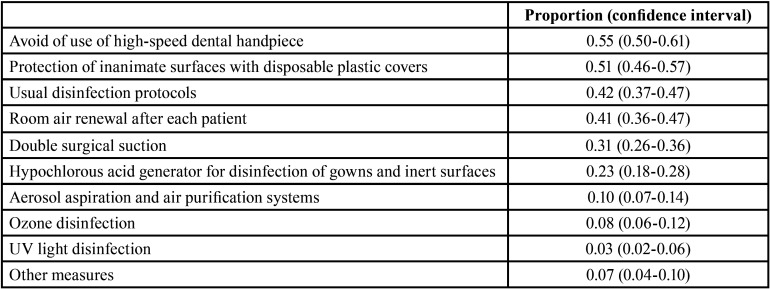
Preventive measures implemented in the dental surgery to avoid cross-infection during the state of alarm (proportion).

Concerning the preventive measures for the patient implemented in the dental surgery, several options could be chosen from a multiple-choice question. The most frequent answer (21.52%) was the combination of preprocedural mouth rinse, hand hygiene, shoe covers, protective goggles and plastic apron.

Regarding questions about Personal Protective Equipment (PPE) used by dentists to treat emergencies during the state of alarm (Table 4), the most used protective elements were face shields (85.17%), FFP2/FFP3 masks (76.69%), surgical caps (76.27%) and nitrile gloves (69.07%).

**Table 4:**
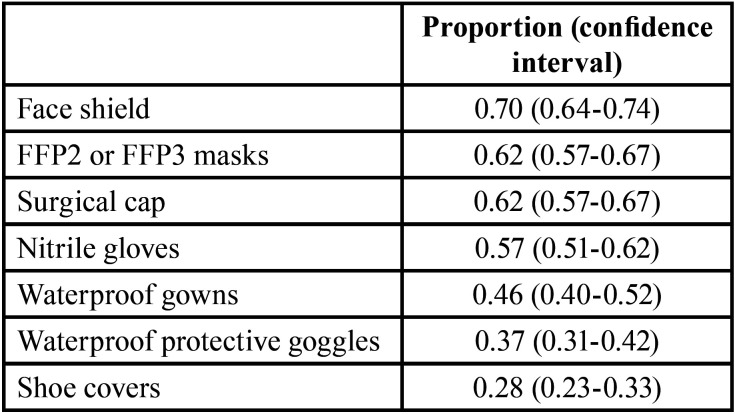
PPE elements used by dentists to treat emergencies during the first state of alarm (proportion).

## Discussion

The main objective of this survey was to show the features of the dental care provision in Spain during the most restrictive period of the first state of al, from 14th March to 4th May 2020. Although only those professionals who had maintained some type of dental emergency care during this time participated in the survey, it could be deduced that a large number of professionals completely ceased their professional activity, as happened in other countries (13,14). The rapid worldwide outbreak of COVID-19 has resulted in considerably psychological stress and fear for healthcare workers, including the fear of getting infected while treating an infected patient, or infecting a family member (13).

The respondents of the survey were mainly women with an age between 30-49 years and general dental practitioners.

One of the most important limitations of our study is the small number of participants, but the sample study group shows similarity with the Spanish General Dental Council Data regarding the population of dentists in Spain nowadays (15): 60.5% female dentists, mean age 41 years and 91% working in private practice.

Most of respondents attended emergencies in person only when it was required, except for those dentists over 60 years of age, who preferred to attend emergencies via telephone. Dentists who continued working attending only emergencies in person were around 75.69%, slightly higher than 60.17% percentage reported by Baracco *et al*. (16). The attitude in older respondents was probably motivated by the high incidence and mortality of COVID-19 in elderly people, with a median age ranging from 51 (17) to 78 (18) years, and the fear that it generates in dentists over 60 years. Our results showed the emergencies attended by telephone were around 19.23%, similar Figures to 25.7% reported by Martínez-Beneyto *et al*. (15).

The majority considered that the number of emergencies had diminished during the state of alarm, which has also been reported in other published results (19), suggesting that COVID-19 strongly influenced people´s dental-care seeking behavior. In addition, we must emphasize that according to our results, there were not so many relevant urgent situations.

Analysis of data identified pain as the most common reason for urgent treatment, which is consistent with the data reported by other studies (19,20). Most telephone attendances were due to pain, especially for those dentists with endodontic expertise or general practitioners. These results coincide with those obtained by Carter *et al*. (21), showing that the main cause of consultations was acute pulpal and periapical complaints.

Most emergencies (59.94%) were solved by telephone, prescribing analgesics, anti-inflammatories or antibiotics. The dental care in Spain, during the peak of pandemic, was mainly remote with 25.7% of dentists attending patients by telephone from home, 25% from health facilities, and 3-5% fulfilling COVID-19 functions, such as telephone triage, as reported by Martínez-Beneyto (15).

Up to 39.18% of dentists stopped working during the pandemic, due to their sense of social responsibility and fear of being carriers of the infection for their relatives (16).

In the case of female dentists, emergency care exclusively via telephone was significant. According to Tysiac-Mista and Dziedzic (14) findings, this may be due to the fact that some women were pregnant or they had to stay at home with their children, because kindergartens and schools were closed because of the pandemic. They also noticed that women were more likely to suspend their clinical practice due to a self-reported feeling of anxiety.

A noTable percentage of respondents (26.60%) attended emergencies in person, being “severe dental pain” the most common reason (69.9%) for urgent face-to-face treatment. These results are in accordance with others previously published (22), highlighting pain of endodontic origin as the most common cause of need for urgent treatment in person.

Among different outstanding measures to limit cross-infection during emergencies care in person is the avoidance of use of high-speed dental handpiece. It is possible when aerosol-generating procedure is implemented, such as dental practice, that 2019-nCoV could possibly spread in airborne transmission (23). When water coolant is combined with bodily fluids in the oral cavity, such as blood and saliva, bioaerosols are created and are commonly contaminated with bacteria, fungi, and viruses, and have the potential to float in the air and be inhaled by the dentists or other patients (20).There is no solid evidence to consistently support that 2019-nCoV in saliva droplets can keep vital along air flow for very long time, but deposition aerosol have been tested positive by Liu *et al*. (24), suggesting that not much vital virus in air flow but tend to deposit to the floor and inanimate surfaces. So, it is advisable to protect inanimate surfaces with disposable covers, as more of the 50% of the respondents do. Air renewal after each patient is another implemented measure recommended, using natural or mechanical ventilation (25,26) and is carried out by more than 40% of those surveyed. However, despite the recommendations regarding COVID-19, a remarkable number of respondents had not changed their protocols related to cross-infection. Several reasons can be argued such as that they considered their disinfection protocols effective enough or that the dental offices were in areas with a low COVID-19 incidence.

Regarding the implemented preventive measures for the patient in dental surgery, most of respondents opted for a combination of the following: preprocedural mouth rinse, hand hygiene, shoe covers, protective goggles and plastic apron. Preprocedural mouth rinse is one of the most effective methods of reducing the proportion of microorganisms in oral aerosol (26). Therefore, preprocedural mouth rinse with 0.2% povidone-iodine or 0.5-1% hydrogen peroxide mouth rinse might reduce the load of corona viruses in saliva (8).

Dentists should follow standard, contact, and airborne precautions including the appropriate use of personal protective equipment (PPE) (8). According to our findings, the most used elements were face shields (used by all dentists working in public healthcare institutions and most of private dentists), FFP2/3 masks, surgical caps and nitrile gloves. The massive use of surgical caps and nitrile gloves are in accordance with the results previously reported by Baracco *et al*. (16), meanwhile the use of FFP2/3 in our study is higher (76.6%) than the percentage recorded in their study. Martínez-Beneyto *et al*. (15) found lack of PPE for dental professionals due to shortage in supplies during the time of their study (one week) during the pandemic peak in April 2020. Differences with our results are probably due to the changed situation in protective equipment supplies over time.

As conclusion, the results of this survey highlight how the situation of dental emergencies were during the first alarm period due to COVID-19 in Spain. Most of respondents attended emergencies in person only when it was required, most of emergencies being solved by prescribing medication by telephone call. There were not many urgent relevant situations, occurring even less than in normal circumstances. The main cause for seeking dental care both, in person and by phone, was dental pain. Dentists had adopted complementary preventive measures to the usual cross-infection preventive protocols, including the avoidance of use of high-speed dental handpiece, room air renewal, patient’s preprocedural mouth rinse and use of PPE.
